# Effects of corn stalk cultivation substrate on the growth of the slippery mushroom (*Pholiota microspora*)

**DOI:** 10.1039/c8ra10627d

**Published:** 2019-02-12

**Authors:** Lingsi Meng, Yongping Fu, Dan Li, Xiaozhong Sun, Yanqi Chen, Xuefei Li, Shuai Xu, Xiao Li, Changtian Li, Bing Song, Yu Li

**Affiliations:** Engineering Research Centre of Chinese Ministry of Education for Edible and Medicinal Fungi, Jilin Agricultural University Changchun 130118 P. R. China song19800123@126.com fungi966@126.com +86-13500881489; Jilin Province Product Quality Supervision Test Institute Changchun 130000 P. R. China

## Abstract

Corn stalks are a major source of agricultural waste in China that have the potential for more efficient utilisation. In this study, we designed substrate formulas with different proportions of corn stalks to cultivate *Pholiota microspora*. The substrate formula for *P*. *microspora* cultivation that could partially or completely replace sawdust with corn stalks was selected through the analysis of mycelial growth rates, fruiting body traits, yield, biological efficiency, nutrients, and mineral composition. Our results showed that the substrate formula T2 (38% wood chips and 38% corn stalks) resulted in the highest yield of 275.66 ± 2.87 g per bag, which was 6.60% higher than that of formula CK, and the highest biological efficiency of 90.75 ± 0.04%, which was 4.58% higher than that of CK, with no significant differences from CK in terms of fruiting body traits, nutrients, or mineral composition. The substrate formula T1 (19% corn stalks) led to mushroom yields with the highest mineral and amino acid contents and was thus more suitable for the cultivation of medicinal *P. microspora*. Therefore, substrates comprising a mixture of corn stalks and sawdust can be used as a novel, inexpensive, and high-yield alternative for the cultivation of *P. microspora*.

## Abstract 2

Corn stalks are a major source of agricultural waste in China that have the potential for more efficient utilisation. In this study, we designed substrate formulas with different proportions of corn stalks to cultivate the *Pholiota microspora*.

## Graphical abstract

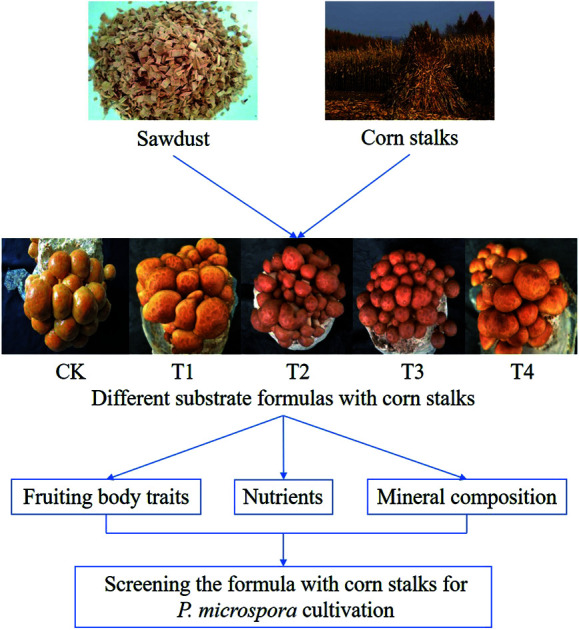

## 1. Introduction

Slippery mushroom (*Pholiota microspora*), also known as *Pholiota nameko*,^1,2^ was originally cultivated in Japan. In the mid-1970s, it was introduced to the southern part of Liaoning Province, China and has become one of main edible fungi cultivated in China.^3^

The “slippery” in the common name of *P. microspora* is named after its surface, which is covered with a layer of smooth and delicious mucus. This mucus has been shown to be beneficial to maintaining energy and brain power in humans and is capable of inhibiting tumours. Research has shown that the mucus is composed of nucleic acids and polysaccharides.^4^ Many find *P. microspora* tasty, and they are nutritious, as they contain crude protein, fat, carbohydrates, crude fibre, calcium, phosphorus, iron, multivitamins, and various amino acids required by the human body. These mushrooms have been shown to demonstrate a number of pharmacological effects, which include anti-aging, antitumour, antibiotic, anti-inflammatory, and immune-boosting properties.^5–7^

Most edible fungi feed on rotten wood and require wood logs and sawdust as the main substrate for cultivation. However, new environmental protection policies and the implementation of a logging ban have resulted in an increasingly short supply of wood materials and soaring wood prices.^8–10^ To better meet the production demand, protect the environment, and save resources, it is important to find alternative substrate materials for mushroom cultivation. The main components of crop stalks are similar to those of wood materials and contain nutrients, such as cellulose, hemicellulose, and lignin, which are required for the growth of edible fungi.^11^ Additionally, large amounts of crop stalks are produced in China and are poorly utilized. Considered a waste product, the stalks are often incinerated, which pollutes the environment and wastes resources. At present, agricultural waste, such as corn stalks, soybean stalks, rice straw, and corn cobs, has been used in the cultivation of edible fungi, such as *Lentinula edodes*, *Pleurotus ostreatus*, *Pleurotus eryngii*, and *Grifola frondosa*.^12–15^ The application of crop stalks in edible fungi cultivation improves the comprehensive utilization of crop waste products and is important for the sustainable development of the environment, energy, and the edible fungus industry.

In this study, two types of agricultural waste, sawdust and corn stalks, were used as raw materials in various proportions to design different cultivation substrate formulas. The effects of different formulas on the yield and growth period of *P. microspora* were investigated using the main agronomic and quality traits as evaluation indexes. The differences in the nutritional compositions of mushrooms grown in different substrates were analysed, and the formula capable of replacing sawdust with corn stalks for *P. microspora* cultivation was selected and optimized, thereby providing a reference for the further development and utilization of agricultural wastes.

## 2. Materials and methods

### 2.1. Strain preparation

The *P. microspora* strain (strain number: CCMJ2806) was obtained from the Engineering Research Center of the Chinese Ministry of Education for Edible and Medicinal Fungi, Jilin Agricultural University. The stock was stored at 4 °C and, after inoculation, cultivated in potato dextrose agar (PDA) at 25 °C in the dark.

The substrate was primarily composed of poplar sawdust and corn stalks and was supplemented with commercially available auxiliary materials, such as wheat bran, cornmeal, soybean meal, lime, and gypsum.

### 2.2. Substrate preparation, inoculation, and culture

Five formulas were designed using poplar sawdust and corn stalks (Table 1). The substrate formula for the cultivation strain CK was 76% poplar sawdust, 15% wheat bran, 5% cornmeal, 2% soybean meal, 1% lime, 1% gypsum, and 58–60% water, with a natural pH. The amounts of corn stalks and poplar sawdust varied in proportion, while the amounts of auxiliary materials remained unchanged.

**Table 1:** Slippery mushroom cultivation formulas (wt%, except for C/N)

Formula	Poplar chips	Corn stalks	Wheat bran	Cornmeal	Soybean meal	Lime	Gypsum	C/N
CK	76	0	15	5	2	1	1	45.93
T1	57	19	15	5	2	1	1	40.55
T2	38	38	15	5	2	1	1	36.07
T3	19	57	15	5	2	1	1	32.29
T4	0	76	15	5	2	1	1	29.06

The substrates (100 g) of each formula were dried in an oven at 60 °C to a constant weight, and the content of carbon and nitrogen were respectively determined using the Kjeldahl method (Kjeltec™ 8000, Foss, Hilleroed, Denmark) and dichromate titration, a chemical method.^15,16^ The carbon to nitrogen ratio (C/N) was then calculated and analysed (Table 1). The tests were performed at the Jilin Province Quality Inspection Institute (Changchun, China). Wheat bran, soybean meal, cornmeal, gypsum, and lime were used as auxiliary materials to provide nitrogen sources and to adjust the pH.^17^

The well-mixed substrate material was placed in a polypropylene bag (17 cm × 33 cm × 0.04 cm, 750 g per bag), autoclaved at 121 °C for 120 min, and then inoculated under aseptic conditions. The culture was incubated at 25 °C in the dark.

### 2.3. Determination of agronomic traits

#### 2.3.1. Mycelial growth and culture cycle

After the hyphae were fully grown, growth was induced at a temperature ranging from 14–18 °C and humidity ranging from 93–96%. During the growth of fruiting bodies, the culture room was intermittently sprayed with water to maintain the following conditions: desired humidity, carbon dioxide (CO_2_) concentration < 2000 ppm, and light at 50–500 lx/12 h. The mycelial growth rate, *i.e.*, the growth rate of mycelium in a given number of days, the time for complete substrate colonization, and the growth period were recorded.

#### 2.3.2. Yield and biological efficiency

When the pilei of the fruiting bodies had not yet unfolded, the fruiting bodies were harvested and weighed using an electronic balance with an accuracy of 0.01 g. The yield of two mushroom harvests was recorded to calculate the biological efficiency (BE) (Table 3). BE (%) = (fruiting body fresh weight/substrate dry weight) × 100%.

The pileus thickness and diameter of the fresh fruiting body as well as the length and diameter of the stipe were measured using a vernier calliper, and the number of fruiting bodies was counted.

### 2.4. Analysis of nutrients

#### 2.4.1. Main nutrients

The fruiting bodies were dried in an oven at 60 °C to a constant weight, and the moisture content of the fruiting bodies was calculated based on the difference in weight before and after drying. The fruiting bodies (100 g) were then pulverized, sifted through a 200-mesh sieve, stored at 4 °C, and sampled for chemical composition analysis. The protein concentration was determined using the Kjeldahl method (Kjeltec™ 8000, Foss, Hilleroed, Denmark),^18^ the fats were analysed using a Soxhlet extractor system (Automatic Fats Analyzer, model 2050, Foss, Hilleroed, Denmark),^19^ and the total sugar was determined according to the Watt and Merrill method using an ultraviolet-visible spectrophotometer (Model T6, New Century, Beijing General Instrument Co., Ltd., Beijing, China).^20^ The tests were performed at the Jilin Province Quality Inspection Institute.

#### 2.4.2. Amino acids

The amino acids of the dried fruiting body powder were analysed using an amino acid analyser (Model Hitachi L8900, Hitachi High Technologies America Inc., Schaumberg, IL, USA).^21^ The tests were performed at the Jilin Province Quality Inspection Institute.

#### 2.4.3. Trace elements and harmful elements

Eight trace elements and four heavy metals common to mushroom fruiting bodies were determined using an inductively coupled plasma mass spectrometer (Model 7700× ICP-MS, Santa Clara, CA, Agilent, USA).^22,23^ The tests were also performed at the Jilin Province Quality Inspection Institute.

## 3. Results and discussion

### 3.1. Effects of corn stalks on mycelium growth and culture period of *P. microspora*

The mycelial growth rate, the time for complete substrate colonization, and the growth period of mushrooms cultivated with different substrate formulas varied significantly (Table 2). The growth rate of mycelia on formula T3 was the highest, followed by that on T2, both of which were significantly higher than that on CK. In terms of the mycelial growth rate, the order of the formulas was T3 > T2 > T1 > CK > T4. The time for complete substrate colonization of mushrooms grown on formula T3 was the shortest, and in terms of time, the order of the formulas was T3 < T2 < T1 < CK < T4. This result indicates that for *P. microspora* grown on substrates supplemented with corn stalks, the growth rate of mycelia is negatively correlated with the time for complete substrate colonization. However, the growth period of the mushrooms grown on formula T2, which was composed of 38% corn stalks, was the shortest. In terms of the growth period, the order of the formulas was T2 < T1 < CK < T3 < T4.

**Table 2:** Mycelial growth rate, yield, and biological conversion of *P. microspora* cultivated on substrates with various proportions of corn stalks^a^

Treatments		CK 76% SD	T1 57% SD, 19% CS	T2 38% SD, 38% CS	T3 19% SD, 57% CS	T4 76% CS
Mycelial growth	GR/mm d^−1^	6.18 ± 0.29c	6.24 ± 0.23c	6.25 ± 0.19b	6.71 ± 0.39a	6.12 ± 0.71c
	TCSC/d	27.4 ± 0.55a	26.6 ± 0.89a	25.80 ± 0.45b	24.8 ± 0.47c	27.8 ± 0.58a
	TIH/d	90.00 ± 0.71c	87.20 ± 0.84b	85.4 ± 0.89d	90.20 ± 0.78c	96.80 ± 1.28a
Yield/g per bag	1st flush	216.03 ± 32.70a	221.47 ± 18.28b	227.15 ± 29.19a	222.20 ± 32.41a	174.00 ± 32.60b
	2nd flush	42.51 ± 9.09a	43.50 ± 8.27ab	44.38 ± 8.94a	33.12 ± 10.25b	22.17 ± 7.07c
	Total	258.59 ± 3.01b	2 6 1.2 ± 5.85c	275.66 ± 2.87a	255.3 ± 6.03b	194.75 ± 6.59d
	BE/%	86.17 ± 0.03b	8 6.96 ± 0.07c	90.75 ± 0.04a	85.14 ± 0.05b	65.29 ± 0.13d

a SD (sawdust), CS (corn straw), GR (growth rate of mycelium), TCSC (time for complete substrate colonization), TIH (time for inoculation to harvest). Different letters (a–d) in the same column and rank indicate significant differences (*P* < 0.05).

Previous studies have shown that the mycelial growth rate in early stages depends mainly on the C/N ratio;^24^ the lower the C/N ratio, the higher the nitrogen content and the faster the mycelium growth. However, too much nitrogen inhibits mycelial growth and delays the formation of fruiting bodies.^25^ Formula T4, which contained only corn stalks, had a C/N ratio of 29.6 and a very low carbon content, whereas the nitrogen content was significantly higher than that of the other formulas. Thus, T4 exhibited the longest time for complete substrate colonization and had the longest mushroom growth period.

### 3.2. Effects of corn stalks on the yield and biological conversion rate of *P. microspora*

**Fig. 1:**
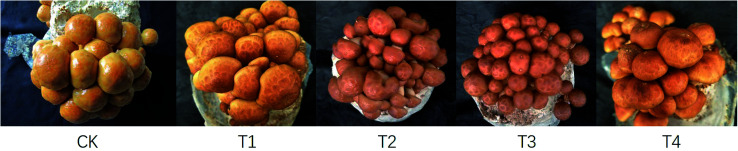
*Pholiota microspora* fruiting in different formulas.

Analysis of the *P. microspora* yields cultivated on substrate formulas with various proportions of corn stalks revealed that as the proportion of corn stalks increased, mushroom yield first increased and then decreased (Table 2 and Fig. 1). The BE and yield of formula T2 were the highest, at 275.66 ± 2.87 g per bag and 90.75 ± 0.04%, respectively, and were higher than those of the control CK by 6.60% and 4.58%, respectively. The BE and yield of T3, which contained 57% corn stalks and 19% wood chips, were similar to those of CK, whereas those of T4, which only contained corn stalks, were the lowest. In terms of yield and BE, the order of the formulas was T2 > T1 > CK > T3 > T4, indicating a negative correlation with the growth period trait.

The yield and BE of T2 were the highest, likely due to an appropriate C/N ratio, which promotes the bioconversion of the substrate and provides more nutrients for the growth of the mushrooms.^15,26^ This result also indicated that as the proportion of corn stalks increased, the C/N ratio gradually decreased and the nitrogen content gradually increased; however, a C/N ratio that was too low reduced the yield and significantly extended the growth period of *P. microspora*.

### 3.3. Effect of corn stalks on fruiting body traits of *P. microspora*

The fruiting body traits of *P. microspora* cultivated on different substrates are shown in Table 3. The stipe length, pileus diameter, and thickness of mushrooms cultivated on T4 were the highest, whereas the stipe width of mushrooms cultivated on T1 was the highest. Although the size of an individual fruiting body of T2 was smaller than that of CK, the number of fruiting bodies of T2 was the highest, and thus, the yield was as well. The ideal commodity traits of *P. microspora* include a small pileus, a short stipe, and a thick pileus.^27^ The commodity traits of the CK were the best, and the fruiting body sizes of T1 or T4 were larger than that of CK, whereas those of T2 and T3 were not significantly different from that of CK. The amount of *P. microspora* mucus decreased as the proportion of corn stalks in the substrate increased, with a gradual decrease of the C/N ratio and a decrease in carbon content. The main components of the mucus are nucleic acids and polysaccharides,^4^ of which carbon is the main constituent element.^28,29^ Therefore, a decrease in carbon inhibits the formation of polysaccharides and nucleic acids in the *P. microspora* mucus. Although the amount of mucus in mushrooms cultivated on T2 slightly decreased, this decrease had little effect on the commodity traits of the mushroom.

**Table 3:** Fruiting body traits of *P. microspora* grown on substrates with various proportions of corn stalks^a^

Treatments	CK	T1	T2	T3	T4
SL (mm)	33.59 ± 0.93c	41.45 ± 2.66b	30.25 ± 2.72cd	29.33 ± 1.61d	49.19 ± 2.43a
SDM (mm)	8.07 ± 1.39bc	9.5 ± 1.76a	6.96 ± 0.9d	8.53 ± 1.23b	7.44 ± 1.06c
PD (mm)	25.32 ± 2.97c	30.37 ± 3.72b	24.63 ± 3.21c	29.92 ± 2.01b	36.97 ± 2.61a
PT (mm)	15.17 ± 1.43b	14.71 ± 1.55c	13.26 ± 1.64d	14.73 ± 1.32c	16.08 ± 1.35a
Quantity	21.34 ± 2.77c	23.11 ± 3.55b	28.07 ± 2.34a	19.65 ± 4.81d	16.49 ± 1.95e
Colour	Yellow	Light yellowish brown	Yellowish brown	Yellowish brown	Dark yellowish brown
Mucus	+++	++	++	+	+

a PT (pileus thickness/mm), PD (pileus diameter/mm), SL (stipe length/mm), SDM (stipe diameter/mm), +(thickness of hyphae). Different letters (a–d) in the same column and rank indicate significant differences (*P* < 0.05).

### 3.4. Effect of corn stalks on the nutritional composition of *P. microspora*

To evaluate the nutritional composition of the *P. microspora* grown on different substrate formulas, the total sugar, fat, protein, and moisture content of the *P. microspora* fruiting bodies grown on different substrates was analysed (Table 4). The total sugar includes the sum of all of the sugar species in the test sample, including reducing and non-reducing sugar species. The analytical results showed that the total sugar content of the mushrooms grown on T4, which contained corn stalks as the sole carbon source, was the highest, whereas those cultivated on other formulas were slightly lower. In particular, the sugar content of mushrooms grown on T2 was not significantly different from those grown on CK. The high total sugar content for T4 was associated with the low moisture content of the fruiting bodies. The crude protein content of mushrooms grown on T3 and CK were high, whereas those of the mushrooms grown on other formulas were slightly lower. Similarly, the fat content of mushrooms grown on T3 and CK were high, whereas those of the mushrooms grown on other formulas were slightly lower, with that for T4 being the lowest. The moisture content of the mushrooms grown on T2 was the highest and exhibited the same trend as the yield variation among the different formulas. The moisture content first increased and then decreased, indicating that the *P. microspora* yield was positively correlated with the moisture content.^30^

**Table 4:** Nutritional composition of *P. microspora* fruiting bodies grown on substrates with various proportions of corn stalks^a^

Treatments	CK	T1	T2	T3	T4
Total sugar%	30.50 ± 0.69b	28.91 ± 0.20c	29.97 ± 0.15b	26.13 ± 0.35d	33.53 ± 0.17a
Fat%	1.15 ± 0.0 8a	0.93 ± 0.16bc	0.82 ± 0.03cd	1.00 ± 0.02ab	0.71 ± 0.04d
Crude protein%	21.04 ± 0.23a	19.95 ± 0.25b	19.93 ± 0.16b	21.19 ± 0.14a	19.22 ± 0.47c
Moisture%	92.06 ± 0.08b	9 2.52 ± 0.28c	93.25 ± 0.23a	89.79 ± 0.31d	87.58 ± 0.34e

a Different letters (a–e) in the same column and rank indicate significant differences (*P* < 0.05).

### 3.5. Effect of corn stalks on the amino acid composition of *P. microspora*

The content of 16 amino acids of fruiting bodies grown on different substrates was analysed, and the results showed that as the proportion of corn stalks increased, the total amino acid content and essential amino acid content first increased and then decreased (Table 5). The total amino acid content of mushrooms grown on various substrate formulas was in the range of 10.76 ± 0.17% to 13.29 ± 0.26%, and the essential amino acid content was in the range of 5.45% to 6.09%. In particular, the total amino acids and essential amino acid content of mushrooms grown on T1 were the highest, at 13.29 ± 0.26% and 6.09 ± 0.06%, respectively, and they were slightly higher than those of mushrooms grown on T2, which were not significantly different from those of mushrooms grown on CK. Among the 16 amino acids, glutamic acid consistently had the highest content for all of the formulas, followed by methionine, whereas the content of glycine, valine, isoleucine, leucine, tyrosine, histidine, and arginine were not affected by the substrate formula variation.

**Table 5:** Amino acid contents (%) and compositions of *P. microspora* fruiting bodies grown on substrates with various proportions of corn stalks^a^

AA	CK	T1	T2	T3	T4
Asp	0.75 ± 0.11c	0.89 ± 0.02a	0.80 ± 0.09b	0.69 ± 0.01d	0.70 ± 0.04d
Thr^b^	0.17 ± 0.01c	0.27 ± 0.02a	0.21 ± 0.02b	0.17 ± 0.01c	0.13 ± 0.01d
Ser	0.05 ± 0.01c	0.13 ± 0.03b	0.10 ± 0.01b	0.30 ± 0.01a	0.04 ± 0.01c
Glu	1.84 ± 0.15ab	1.93 ± 0.07a	1.75 ± 0.05b	1.25 ± 0.07d	1.67 ± 0.27bc
Gly	0.42 ± 0.02b	0.51 ± 0.04a	0.46 ± 0.01ab	0.43 ± 0.01b	0.40 ± 0.01bc
Ala	0.39 ± 0.01c	0.50 ± 0.01a	0.42 ± 0.01b	0.31 ± 0.01d	0.34 ± 0.01cd
Val^b^	0.779 ± 0.04a	0.81 ± 0.02a	0.75 ± 0.03ab	0.7 ± 0.03ab	0.73 ± 0.05b
Met^b^	1.57 ± 0.10b	1.60 ± 0.17b	1.76 ± 0.16a	1.29 ± 0.11c	1.41 ± 0.18bc
Ile^b^	0.57 ± 0.02ab	0.59 ± 0.03a	0.53 ± 0.02b	0.579 ± 0.02ab	0.53 ± 0.02b
Leu^b^	1.25 ± 0.12a	1.18 ± 0.21ab	1.24 ± 0.14a	1.249 ± 0.08a	1.17 ± 0.31ab
Tyr	0.33 ± 0.01ab	0.37 ± 0.02a	0.32 ± 0.01ab	0.31 ± 0.01b	0.29 ± 0.01b
Phe^b^	0.72 ± 0.03b	0.83 ± 0.03a	0.68 ± 0.03c	0.70 ± 0.02b	0.69 ± 0.02bc
Lys^b^	0.83 ± 0.04a	0.81 ± 0.3b	0.85 ± 0.03a	0.84 ± 0.05a	0.78 ± 0.16bc
His	0.35 ± 0.02a	0.37 ± 0.02a	0.36 ± 0.01a	0.34 ± 0.01ab	0.31 ± 0.01b
Arg	0.99 ± 0.04a	0.86 ± 0.03b	0.97 ± 0.08a	0.94 ± 0.04a	0.93 ± 0.11ab
Pro	0.73 ± 0.01bc	1.65 ± 0.09a	0.79 ± 0.02b	0.82 ± 0.02b	0.64 ± 0.04c
Total essential amino acids	5.88 ± 0.08b	6.09 ± 0.06a	6.02 ± 0.03a	5.58 ± 0.09b	5.45 ± 0.04bc
Total amino acids	11.72 ± 0.14b	13.29 ± 0.26a	11.98 ± 0.25b	10.97 ± 0.23c	10.76 ± 0.17c

a Different letters (a–d) in the same column and rank indicate significant differences (*P* < 0.05).

b Essential amino acids.

### 3.6. Effect of corn stalks on the mineral composition of *P. microspora*

Edible fungi are prone to accumulating heavy metals, and the mineral composition of the fruiting bodies has a major impact on the quality of the product.^31,32^ In this study, we analysed the amount of trace elements and harmful metal elements in the fruiting bodies grown on different formulas and found that the mineral composition was greatly affected by the different cultivation substrates (Table 6). Regarding the essential major elements, such as calcium, magnesium, and sodium, T1 and T3 both had higher amounts of calcium and magnesium than CK. The fruiting bodies grown on T2 had a higher sodium content and lower calcium and magnesium content than those grown on CK. In particular, the calcium content of fruiting bodies grown on T2 was 579.08 ± 7.42 mg kg^−1^, which was significantly lower than that of the fruiting bodies grown on other formulas. It was found that calcium facilitates the formation of fruiting bodies,^33^ suggesting that the small size of the fruiting bodies grown on T2 was due to the low calcium level of the formula. The amount of trace elements, such as manganese, iron, zinc, copper, and selenium, which are beneficial to the human body,^34,35^ were higher in mushrooms grown on corn stalk formulas than in those grown on CK, except for the manganese and selenium content of mushrooms grown on T2, which were slightly lower than those of mushrooms grown on CK.

**Table 6:** Mineral composition of *P. microspora* fruiting bodies grown on substrates with various proportions of corn stalks^a^

Treatments		CK	T1	T2	T3	T4
Major element	Calcium/mg kg^−1^	820.33 ± 1.53c	1250.31 ± 3.05a	579.08 ± 7.42e	780.65 ± 1.54d	870.52 ± 3.36b
	Sodium/mg kg^−1^	140.33 ± 2.52d	1 4 1.00 ± 3.12d	160.34 ± 1.53c	213.01 ± 4.07b	231.33 ± 11.06a
	Magnesium/mg kg^−1^	1220.12 ± 1.78b	1351.77 ± 4.59a	980.13 ± 4.25c	1369.89 ± 3.18a	1240.55 ± 5.76b
Trace element	Manganese/mg kg^−1^	18.34 ± 1.06c	29.37 ± 2.41b	17.19 ± 0.98c	25.30 ± 1.52b	32.03 ± 0.44a
	Iron/mg kg^−1^	130.29 ± 1.21c	182.10 ± 4.34a	133.71 ± 0.28c	171.69 ± 5.72ab	170.33 ± 3.57b
	Copper/mg kg^−1^	12.78 ± 2.99c	16.23 ± 1.79b	17.24 ± 2.57a	14.11 ± 0.37c	16.94 ± 2.44a
	Zinc/mg kg^−1^	51.44 ± 1.34c	55.78 ± 2.82a	53.09 ± 3.66ab	49.37 ± 1.24c	55.24 ± 3.11a
	Selenium/mg kg^−1^	0.32 ± 0.01b	0.54 ± 0.08a	0.22 ± 0.02c	0.34 ± 0.01b	0.23 ± 0.03c
Harmful element	Arsenic/mg kg^−1^	1.12 ± 0.08c	0.83 ± 0.04d	1.41 ± 0.06a	0.52 ± 0.05e	1.25 ± 0.08b
	Cadmium/mg kg^−1^	0.31 ± 0.02c	0.52 ± 0.05a	0.38 ± 0.03b	0.41 ± 0.03b	0.42 ± 0.03b
	Mercury/mg kg^−1^	0.03 ± 0.01b	0.03 ± 0.01b	0.03 ± 0.01a	0.03 ± 0.01b	0.03 ± 0.01b
	Lead/mg kg^−1^	0.92 ± 0.01d	2.39 ± 0.03b	1.49 ± 0.01c	2.59 ± 0.08b	3.64 ± 0.41a

a Different letters (a–e) in the same column and rank indicate significant differences (*P* < 0.05).

The amount of elements, such as arsenic, chromium, lead, and mercury, which are harmful to the human body, have a significant impact on the production of edible fungi.^36–39^ In this study, we found that the arsenic, cadmium, and lead content in fruiting bodies grown on corn stalk-added formulas were all higher than those in mushrooms grown on CK (sawdust only), whereas there were no differences in the mercury content of mushrooms grown on different formulas. All amounts of harmful elements met China's food safety standards for edible fungi.^40^ The slightly high arsenic, cadmium, and lead content of the fruiting bodies grown on corn stalk-added formulas is likely attributed to the higher content of heavy metals in the corn stalk substrate than in the wood chip substrate.

## 4. Conclusions

The results show that corn stalks can partially replace sawdust as a practical and easily available substrate for *P. microspora* cultivation. The corn stalk-added formula T2 (38% SD + 38% CS) significantly increased the yield and biological efficiency and shortened the growth period of the mushroom without affecting the commodity traits or nutritional composition of the fruiting bodies compared to mushrooms grown on the CK. Considering environmental protection policies and the recycling of agricultural waste, formula T2 is worth utilizing. In comparison, the total amino acid and trace element content as well as the yields of mushrooms grown on T1 (19% CS) were high, making T1 an ideal substrate formula for the cultivation of medicinal *P. microspora*.

## Authors' contributions

LSM, YPF and DL conceived and designed the experiments, performed the experiments, analysed the data, wrote the paper, and prepared the figures and/or tables; these authors contributed equally to this work. YQC, XFL, XZS, CTL and XL conceived and designed the experiments. BS and YL conceived and designed the experiments, analysed the data, contributed reagents/materials/analysis tools, wrote the paper, prepared figures and/or tables, and reviewed drafts of the paper. All authors read and approved the final manuscript.

## Conflicts of interest

The authors declare that they have no conflict of interest.

## Abbreviations

BE: Biological efficiency
PDA: Potato dextrose agar
CO_2_: Carbon dioxide
C/N: Carbon to nitrogen ratio
SD: Sawdust
CS: Corn straw
GR: Growth rate of mycelium
TCSC: Time for complete substrate colonization
TIH: Time for inoculation to harvest
PT: Pileus thickness/mm
PD: Pileus diameter/mm
SL: Stipe length/mm
SDM: Stipe diameter/mm

## Supplementary Material
